# RNA Quality in Post-mortem Human Brain Tissue Is Affected by Alzheimer’s Disease

**DOI:** 10.3389/fnmol.2021.780352

**Published:** 2021-12-21

**Authors:** Blake Highet, Remai Parker, Richard L. M. Faull, Maurice A. Curtis, Brigid Ryan

**Affiliations:** ^1^Department of Anatomy and Medical Imaging, Faculty of Medical and Health Sciences, University of Auckland, Auckland, New Zealand; ^2^Centre for Brain Research, Faculty of Medical and Health Sciences, University of Auckland, Auckland, New Zealand

**Keywords:** RNA, human, brain, Alzheimer’s disease, Parkinson’s disease, Huntington’s disease

## Abstract

Gene expression studies of human *post-mortem* brain tissue are useful for understanding the pathogenesis of neurodegenerative disease. These studies rely on the assumption that RNA quality is consistent between disease and neurologically normal cases; however, previous studies have suggested that RNA quality may be affected by neurodegenerative disease. Here, we compared RNA quality in human *post-mortem* brain tissue between neurologically normal cases (*n* = 14) and neurodegenerative disease cases (Alzheimer’s disease *n* = 10; Parkinson’s disease *n* = 11; and Huntington’s disease *n* = 9) in regions affected by pathology and regions that are relatively devoid of pathology. We identified a statistically significant decrease in RNA integrity number (RIN) in Alzheimer’s disease tissue relative to neurologically normal tissue (mixed effects model, *p* = 0.04). There were no statistically significant differences between neurologically normal cases and Parkinson’s disease or Huntington’s disease cases. Next, we investigated whether total RNA quality affected mRNA quantification, by correlating RIN with qPCR threshold cycle (C_T_). C_T_ values for all six genes investigated were strongly correlated with RIN (*p* < 0.05, Pearson correlation); this effect was only partially mitigated by normalization to RPL30. Our results indicate that RNA quality is decreased in Alzheimer’s disease tissue. We recommend that RIN should be considered when this tissue is used in gene expression analyses.

## Introduction

Gene expression studies in human *post-mortem* brain tissue are useful tools for understanding the pathogenesis of neurodegenerative disease. It is critical to obtain high-quality RNA from human *post-mortem* brain tissue, in order to enable transcriptomic analyses in health and disease (Fleige and Pfaffl, 2006). Human brain banks hold vast collections of archived frozen tissue from both neurologically normal people and those who died with neurological disease. However, the RNA quality of these samples varies significantly and can preclude some RNA analyses. In contrast to animal studies, it is not possible to control all *pre-mortem* and *post-mortem* variables when collecting human brain tissue. Recent studies have identified several factors related to the collection, processing, and storage of human brain tissue that may contribute to RNA quality (Birdsill et al., 2011; Clement et al., 2016; White et al., 2018).

One *pre-mortem* variable that may affect RNA quality in human brain tissue is neurodegenerative disease. For example, it is well-established that Alzheimer’s disease (AD) is associated with oxidative stress, which can damage RNA species (Nunomura et al., 2006). Further, agonal state has been linked to RNA quality (Tomita et al., 2004; Chevyreva et al., 2008; White et al., 2018), so if agonal state varies systematically between disease and control groups, this would be expected to affect RNA quality. Research to date is equivocal: one study has reported that RNA quality is lower in AD cortex and cerebellum compared to neurologically normal cases (White et al., 2018), while two studies have reported no difference in AD cerebellum (Chevyreva et al., 2008; Birdsill et al., 2011), Parkinson’s disease (PD) cerebellum (Chevyreva et al., 2008; Birdsill et al., 2011), or Huntington’s disease (HD) cerebellum (Chevyreva et al., 2008). Determining whether RNA quality varies with disease is vital, because methods used to quantify changes in RNA expression in disease relative to neurologically normal controls assume that RNA quality does not differ systematically between these two groups. If RNA quality is affected by pathology, we would expect RNA quality to be reduced in affected brain regions relative to neurologically normal cases, but to be unchanged in brain regions that are relatively devoid of pathology.

The aim of this research was to determine whether RNA quality in human *post-mortem* brain tissue differs by disease. We compared neurologically normal cases to those with AD, PD, or HD. This study utilized a total of 107 RNA preparations from 44 different cases. In all disease cases RNA was extracted from one affected region [AD: hippocampus (HP); PD: substantia nigra (SN); HD: caudate nucleus (CN)] and one relatively unaffected region: cerebellum (CB). RNA was extracted from these same regions in neurologically normal cases for comparison of RNA quality.

## Method

### Human Tissue Collection and Preparation

*Post-mortem* human brain tissue was obtained from the Neurological Foundation Human Brain Bank at the University of Auckland. Human tissue was donated to the Brain Bank with consent from donors’ families and its use in this project was approved by the University of Auckland Human Participants Ethics Committee (011654). Tissue from 14 neurologically normal cases, 10 AD cases, 11 PD cases, and 9 HD cases was included in the study. Details of diagnosis, age, sex, cause of death, and *post-mortem* delay are available in Supplementary Table 1. No statistically significant differences in the age at death were observed between disease groups in any regions. Normal cases had no previous history of neurological disorder and cause of death was unrelated to any neurological condition. Pathological assessment of normal cases reported no disease-related pathology beyond that expected in aged individuals, and clinical diagnosis of disease cases were confirmed through pathological examination of disease-specific protein aggregates by a neuropathologist.

The brain regions used in this study were: CB (all cases); HP (normal and AD); SN (normal and PD); and CN (normal and HD). The CB was chosen as a region that is relatively devoid of pathology in these neurodegenerative diseases (as demonstrated by pathology reports for all cases). All other regions were chosen as areas with high pathology in their respective diseases. Disease cases used only displayed disease-specific pathology to avoid confounding results with mixed pathologies or other comorbidities.

Brains were processed and stored according to previously published protocols (Waldvogel et al., 2006). Briefly, the brain was hemi-dissected and the left hemisphere was dissected into previously defined tissue blocks. These blocks were frozen immediately on powdered dry ice, double-wrapped in aluminum foil, and stored at −80°C until required for RNA extraction. Blocks were stored for a period ranging from 1 to 26 years. Sections (20 μm) of fresh-frozen blocks were cut using a cryostat (Leica CM30505), placed in chilled RNase-free Eppendorf microtubes, weighed on a microscale (6–21 mg per sample) and stored at −80°C. Note that tissue blocks were chosen based on limited freeze-thaw cycles, as this has been shown to greatly impact RNA quality (Sherwood K.R. et al., 2011). Blocks that had been previously sampled from more than once (and therefore were at greater risk of being thawed and re-frozen) were not included.

### RNA Extraction

Total RNA was extracted from fresh-frozen human brain tissue sections using the RNeasy Mini Kit (QIAGEN) according to manufacturer’s instructions with pre-processing using a recently published protocol (Highet et al., 2021). Tissue was placed in a pre-chilled Eppendorf tube with 300 μL of lysis buffer and disrupted using pre-chilled plastic pestles. An additional 300 μL of lysis buffer was then added before homogenizing the sample using needle and syringe (21 g followed by 25 g). Total RNA was eluted in 30 μL RNase-free water. To minimize RNA degradation, all materials and work surfaces were cleaned with RNaseZap (Sigma-Aldrich). RNA concentration (ng/μL) and purity (A260/280) were determined using spectrophotometry (Nanodrop 1000, Thermo Fisher), and RNA yield per input weight (μg/mg) was estimated by dividing the yield [concentration (ng/μL) times total sample volume (30 μL)] by the weight of the tissue (mg). Mean A260/280 was 2.0 (range 1.3–2.4). A260/280 was between 1.9 and 2.1 in 102/107 samples. RNA integrity (RNA Integrity Number; RIN) was assessed using the Agilent RNA 6000 Nano Kit (Agilent Technologies) on an Agilent 2100 Bioanalyzer (Agilent Technologies). Our RNA quality was similar to that previously reported from other human brain banks: 80.4% of tissue specimens had a RIN > 6, similar to that reported by White et al. (2018) (83% of 1,068 specimens in the NIH NeuroBioBank had RIN > 6).

### Quantitative PCR

Total RNA was diluted to a concentration of 25 ng/μL prior to cDNA synthesis to ensure that total RNA input was consistent across samples. Total RNA was DNase-treated prior to cDNA synthesis (RQ1 RNase-Free DNase, Promega). 225 ng of total RNA from each sample was used to generate cDNA using the High-Capacity RNA-to-cDNA Kit (Applied Biosystems), which includes a combination of random octamers and oligoDT to minimize the impact of partial RNA degradation on downstream assays. cDNA was diluted 1 in 5 in DPEC-treated water before subsequent use for qPCR on a 7900HT Fast Real-Time PCR machine (Applied Biosystems). The reaction volume was 10 μL. Each reaction included: 1 μL cDNA, 5 μL Platinum^®^ SYBR^®^ Green RT-qPCR SuperMix-UDG with Rox (Life Technologies), 1.2 μL primer mix (3 μM forward primer and 3 μM reverse primer; final concentration 180 nM), and 2.8 μL DPEC-treated water. Reactions were prepared in clear qPCR 384-well plates (MicroAmp™ Optical 384-Well Reaction Plate, ThermoFisher) sealed with adhesive covers. All amplifications were carried out in triplicate. The qPCR cycling conditions were as follows: 2 min at 50°C to incubate UDG, 2 min at 95°C to activate DNA polymerase, followed by 40 cycles of denaturing at 95°C for 15 s, and annealing and extension at 60°C for 60 s. The melt curve protocol followed with 15 s at 95°C, 15 s at 60°C, and 15 s at 95°C. C_T_ was determined automatically using QuantStudio 12K Flex Software v.1.3.

Six genes were chosen to examine the effects of RIN on mRNA quantification. The genes were selected based on their use in previous publications quantifying mRNA expression in human brain tissue (Coulson et al., 2008; Penna et al., 2011; Rydbirk et al., 2016; Highet et al., 2021). Details for the primers used in this study are available in Supplementary Table 2. For one gene (*POLR2A*) we used two primer pairs of differing amplicon length (73 bp vs. 131 bp) to determine whether shorter amplicon length mitigated the effect of moderate RNA degradation on mRNA quantification (Fleige and Pfaffl, 2006; Coulson et al., 2008). Primer efficiencies were determined for all primers by performing qPCR using serial dilutions of cDNA (standard curve method) from one case (H177 CB) and were all between 95 and 108% (*r*^2^ > 0.99). All primers produced melt curves with single peaks indicating amplification of a single, specific product. All no template control reactions and no reverse transcription control reactions produced C_T_ values that were “undetermined” or <5 C_T_ higher than all template reactions. ΔC_T_ (Livak and Schmittgen, 2001) was determined relative to *RPL30*.

### Statistical Analyses

Differences in RIN and yield across regions and between groups (disease vs. neurologically normal) were determined using a mixed effects model approach to analyze repeated measures data, because there were missing values. The Sidak method was used to correct for multiple comparisons. Unless stated otherwise, values reported represent mean values for each group and region. *A priori* power analysis using G*Power 3 (Faul et al., 2007) indicated that a minimum sample size of 8 cases per group was required (effect size = 0.4, power = 0.8, α = 0.05). To compare variables between the four brain regions studied in neurologically normal cases, a one-way ANOVA was conducted. Linear correlations between two numerical variables were tested using Pearson’s correlations, with 0.3 ≤ *r* ≤ 0.5 and −0.5 ≤ *r* ≤ 0.3 considered weak, *r* = 0.5–0.7 and *r* = −0.5 to −0.7 considered moderate, and *r* ≥ 0.7 and *r* ≤ −0.7 considered strong correlations. Statistical significance was set at *p* < 0.05. Significance is indicated by *p < 0.05, ^**^p < 0.01, ^***^p < 0.001, and ^****^p < 0.0001. All statistical testing was performed using GraphPad Prism 8.0.0.

## Results

### RNA Integrity Number Is Decreased in Alzheimer’s Disease

**FIGURE 1:**
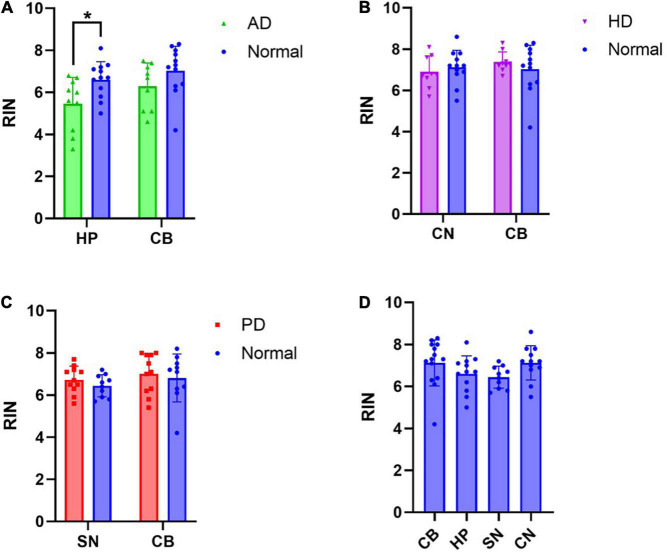
RNA integrity number (RIN) is decreased in Alzheimer’s disease hippocampus: RIN values from RNA extracted from disease affected and unaffected regions were compared between disease groups and neurologically normal cases, as well as across all regions of neurologically normal cases. **(A)** A significant decrease in RIN was observed in the AD HP. **(B)** No significant differences in RIN were observed in the CN or CB in HD. **(C)** No significant differences in RIN were observed in the SN or CB in PD. **(D)** RIN did not differ by region in normal cases. *p < 0.05.

To determine whether RIN is affected by disease, comparisons in RIN values of RNA extracted from affected and unaffected brain regions were made between neurologically normal and disease cases (Figure 1). A mixed effects model was performed to analyze the effect of disease and region on RIN. Simple main effects analysis showed that in AD, both disease (*p* = 0.0350) and region (*p* = 0.0142) had a statistically significant effect on RIN. However, there was not a statistically significant interaction between the effects of disease and region (*p* = 0.3914). Sidak’s test for multiple comparisons found that the mean RIN was statistically significantly lower in AD HP than in neurologically normal HP (Figure 1A: normal HP: 6.60, AD HP: 5.45, *p* = 0.0380). The difference in mean RIN between AD CB and neurologically normal CB did not reach statistical significance (Figure 1A: Normal CB: 6.95, AD CB: 6.14, *p* = 0.1845). In contrast, there was no effect of disease or region on RIN in HD (Figure 1B: normal CN: 7.125, HD CN: 6.9, *p* = 0.808. Normal CB: 7.13, HD CB: 7.35, *p* = 0.8405). RIN was also unchanged in either the SN or CB in PD (Figure 1C: normal SN: 6.44, PD SN: 6.73, *p* = 0.6006. Normal CB: 7.12, PD CB: 7.01, *p* = 0.9439). Furthermore, RIN did not differ by region in normal cases (Figure 1D: One-way ANOVA, *p* = 0.1724). Therefore, these results suggest that RNA extracted from AD tissue is significantly degraded compared to RNA from neurologically normal tissue.

Next, we excluded cases from the analysis if they exhibited RIN values < 5 (3x AD cases, 1x normal case excluded), as this is a commonly used cut-off for acceptable RNA quality (Fleige and Pfaffl, 2006). In this analysis, there was no statistically significant difference in RIN between normal and AD HP tissue (Sidak’s test for multiple comparisons *p* = 0.3948) and all other comparisons remained insignificant (Supplementary Figure 1). This result supports the recommendation that cases with RIN < 5 should be excluded from qPCR analyses.

To establish whether RNA yield is altered in affected brain regions, comparisons in RNA yield per input tissue weight extracted from affected and unaffected brain regions were made between normal and disease cases (Supplementary Figure 2). No significant differences were observed in RNA yield for any brain regions across all diseases (Supplementary Figures 2A–C). However, unlike RIN, RNA yield differed by region in normal cases (Supplementary Figure 2D). These differences in region between normal cases likely reflected regional differences in the proportion of white matter in the tissue. Therefore, these differences can be mitigated by using equal RNA input for cDNA synthesis and normalizing to a suitable housekeeping gene.

### RNA Integrity Number Is Correlated With C_T_

**FIGURE 2:**
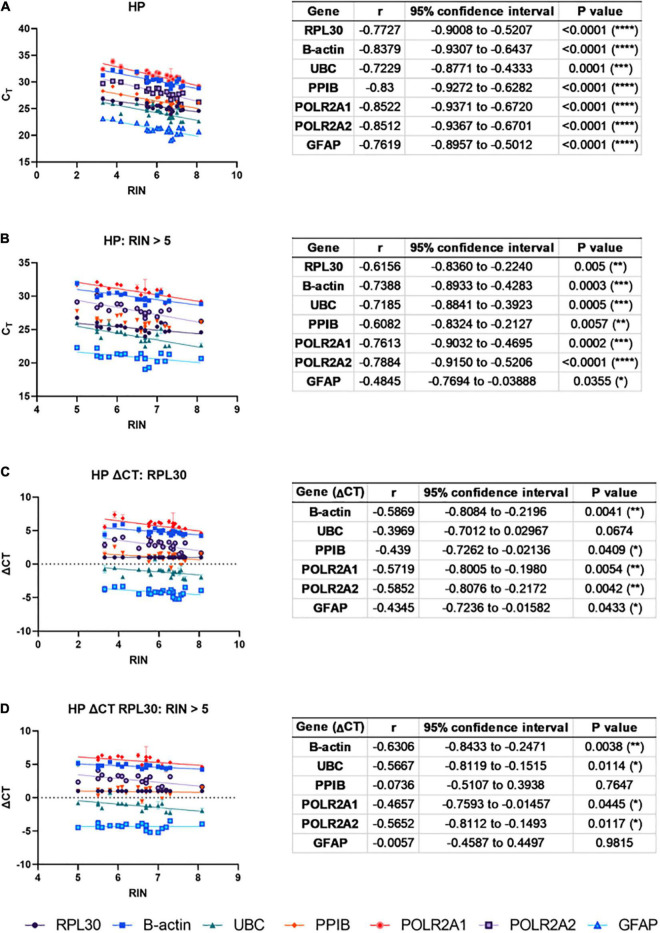
RNA integrity number (RIN) is correlated with CT: To ascertain whether degraded total RNA affects mRNA quantification using qPCR, correlations between RIN and qPCR CT for six genes commonly used in qPCR studies of human brain tissue were made across pooled normal and AD HP cases. **(A)** Strong, negative correlations were observed between CT and RIN for all genes. **(B)** After excluding RIN values < 5, strong negative correlations were still observed for three out of six genes (*B-actin, UBC, POLR2A*), moderate negative correlations were observed for two out of six genes (*RPL30, PPIB*), and *GFAP* showed a weak, negative correlation. To mitigate this, CT values were normalized to *RPL30* using the ΔCT method. **(C)** Moderate, negative correlations were still observed in two out of five genes (*B-actin, POLR2A*) and weak negative correlations were observed in two out of five genes (*UBC, PPIB*). **(D)** Removing RIN values < 5 still produced moderate, negative correlations for two out of five genes (*B-actin, UBC*), and one gene showed a moderate or weak negative correlation depending on the primer pair used (*POLR2A*).

Next, to ascertain whether degraded total RNA affects mRNA quantification using qPCR, correlations between RIN and qPCR C_T_ for six genes commonly used in qPCR studies of human brain tissue were made across pooled normal and AD HP cases (Figure 2). Strong negative correlations were observed between raw C_T_ and RIN values for all genes (Figure 2A). Next, cases were removed from the analysis if they exhibited RIN values < 5 (3x AD cases excluded). This improved the results; however, strong negative correlations were still observed for 3/6 genes; moderate negative correlations were observed for 2/6 genes, and a weak negative correlation was observed for *GFAP* (Figure 2B). Similar results were observed in the cerebellum (Supplementary Figures 3A,B). To determine whether this relationship could be mitigated by normalization to a housekeeping gene, C_T_ values for the remaining five genes were normalized to *RPL30* using the ΔC_T_ method before reconducting correlations with RIN. This improved on the raw C_T_ results; however, moderate negative correlations were still observed for 2/5 genes and weak negative correlations were observed for 2/5 genes (Figure 2C). Therefore, cases were again removed from analysis if they contained RIN values < 5 (Figure 2D). Again, this improved the results: two genes displayed moderate negative correlations (*B-actin*, *UBC*), one gene displayed a weak or moderate negative correlation depending on the primer pair used (*POLR2A*), and two genes showed no correlation (*PPIB* and *GFAP*). Therefore, RIN is correlated with C_T_ for some genes even after normalization to *RPL30* using the ΔC_T_ method and removal of cases with low RIN values, indicating that degraded RNA affects results in downstream applications relying on quantification of RNA signal. This suggests that cases must be matched for RIN when analyzing disease differences, or that RIN must be considered as a co-variate during statistical analysis. Similar correlations were found for *POLR2A* using primers with both shorter (73 bp) and longer (131 bp) amplicon lengths. Note that no significant correlations were observed when this normalization was performed in the CB (Supplementary Figure 3C); however, CB samples contained higher average RIN values compared to HP samples.

To determine whether age at death, *post-mortem* delay or storage time affects RNA integrity, correlations between both RIN and RNA yield and these factors were conducted (Supplementary Figure 4). These correlations were conducted using CB RNA pooled across all cases and diseases, to maximize the number of independent samples. A moderate, negative correlation was observed between age at death and RIN (Supplementary Figure 4A), but no correlations were observed between either *post-mortem* delay or storage time with RIN (Supplementary Figures 4B,C). No correlations were observed between RNA yield and any of these factors (Supplementary Figures 4D–F). These results suggest that RIN decreases with age and highlights the importance of age-matching when conducting comparisons between cases.

## Discussion

Overall, our results show that RNA quality is lower in AD tissue compared to neurologically normal tissue. Importantly, the decrease in RNA quality in the HP was small (∼1 RIN) and this effect was lost when cases with RIN < 5 were excluded. Nonetheless, we show that RIN strongly correlates with RT-qPCR C_T_ and that normalizing to a housekeeping gene using the ΔC_T_ method did not ameliorate this correlation for all genes, even when cases with RIN < 5 were excluded. These findings highlight that caution must be taken when conducting mRNA quantification studies comparing AD human brain tissue to neurologically normal tissue.

Our finding of decreased RNA quality in the AD brain is consistent with a previously published study (White et al., 2018). We did not find a statistically significant interaction between the effect of disease and region, suggesting that the effect of disease does not differ by region. This result suggests that decreased RNA quality in AD is not restricted to regions with high pathology, given that the CB is relatively devoid of pathology. Two previous studies reported that there was no change in RNA quality in the AD CB (Chevyreva et al., 2008; Birdsill et al., 2011). It should be noted that White et al. (2018) did report a decrease in RNA quality in the AD CB and that their sample size (*n* = 74 AD) was much larger than both the current study and the studies mentioned above (*n* = 7 – 26 AD). Therefore, it cannot be ruled out that the smaller studies were underpowered to detect small decreases in RNA quality in the AD CB.

In contrast to AD, we found no significant difference in RNA quality in PD or HD in either affected regions or the CB. This result is also consistent with previous studies that reported no changes in RNA quality in cortical tissue (White et al., 2018) or CB (Chevyreva et al., 2008; Birdsill et al., 2011) in these diseases, suggesting that pathology effects on RNA quality are specific to AD and not to neurodegenerative diseases in general. However, small changes in RNA quality in these diseases may yet be identified given no study has looked at a sample size greater than 16 subjects in either of these diseases.

There are several implications of lower RNA quality in AD tissue on downstream qPCR studies. One possibility is that lower RIN reflects degradation of only ribosomal RNA subunits, but not mRNA, meaning that lower RNA quality would not influence gene expression analyses. To investigate this, we correlated RIN with mRNA expression levels (as measured by C_T_). We found that RIN showed strong, negative correlations for all six genes investigated, including commonly used housekeeping genes. This result is consistent with correlations between RIN and C_T_ observed in bovine tissue (Fleige and Pfaffl, 2006), suggesting that low RIN does reflect mRNA degradation. It also supports the hypothesis that a false down-regulation of gene expression will be obtained if RNA degradation is higher in pathological samples (Barrachina et al., 2006).

To accommodate this global mRNA degradation in AD tissue, normalizing to a housekeeping gene using the ΔC_T_ method should mask lower global mRNA levels in AD (Fleige and Pfaffl, 2006). However, we still found significant correlations in all genes investigated when normalized to *RPL30*, although the strength of these correlations was weaker. This result is inconsistent with other studies utilizing this method (Fleige and Pfaffl, 2006), and suggests that the ΔC_T_ method relies on global degradation affecting the gene of interest and the housekeeping gene to the same extent. To mitigate the effect of moderate RNA degradation on qPCR analyses, previous studies have recommended including random primers in the cDNA synthesis reaction and designing primers to produce amplicons of < 250 base pairs (Fleige and Pfaffl, 2006; Coulson et al., 2008). Although both approaches were employed in the current study, correlations between RIN and C_T_/ΔC_T_ were still identified, suggesting that these strategies alone are not adequate for mitigating the effect of RNA degradation.

To avoid these issues, the simplest proposed solution is to match groups based on RIN (Tunbridge et al., 2011; White et al., 2018). However, when we removed three AD cases with RIN < 5 from our analyses and matched groups by RIN, three out of five genes investigated still displayed significant correlations when normalized to *RPL30*. This result corroborates a recent RNA-seq study which found that RIN affected differential gene expression analysis of AD HP tissue compared to normal HP tissue even with a RIN cut-off of 7 (Crist et al., 2021). Thus, this study and others have recommended that RIN is included as a co-variate during statistical analyses (Crist et al., 2021; Guennewig et al., 2021).

There are a number of possible factors that may be driving the observed RNA degradation in AD brain tissue. Our finding that there is no difference in the effect of disease by region indicates that RNA degradation in AD is not restricted to regions that are most affected by pathology. Our finding of an effect in AD, but not PD or HD, suggests that RNA degradation is specific to AD, rather than being a feature of neurodegenerative disease in general. Previous studies have reported a correlation between tissue pH and RIN (Webster, 2006; Chevyreva et al., 2008), and that pH is decreased in AD brain tissue relative to neurologically normal controls (Decker et al., 2021). It is possible therefore, that the effect of disease on RIN is mediated by pH. However, White et al. (2018) reported that there was no relationship between RIN and pH in cortex or CB in a large cohort of 789 cases. Prolonged agonal state has also been associated with decreased RNA integrity and has been suggested to decrease pH (Tomita et al., 2004). It is possible that the cause of death in neurologically normal cases tends to be more sudden than the protracted course of AD cases, meaning that RNA degradation is affected by agonal factors rather than AD itself. However, the relationship between agonal state and RIN has been challenged (Sherwood K. et al., 2011), and prolonged agonal state would also be expected in other neurodegenerative disease. Further work to elucidate the mechanism of RNA degradation in AD brain tissue is warranted.

We cannot discount the possibility that age at death may have contributed to the decreased RIN observed in AD tissue. Although the difference in mean age at death between AD and neurologically normal cases was not statistically significant, the mean age at death was slightly lower in neurologically normal cases. In CB tissue across all cases included in this study, we found a moderate inverse correlation between age at death and RIN, so it is possible that our finding of decreased RIN in AD cases was partially influenced by age at death. In this study we prioritized case selection based on minimum freeze-thawing, so selecting paired cases based on age at death was not possible. This observation underlines the importance of matching cases by age at death wherever possible, or including age at death as a covariate.

## Conclusion

In conclusion, the current study has demonstrated the importance of considering RNA degradation when quantifying gene expression in human *post-mortem* brain tissue, particularly for studies comparing AD tissue to neurologically normal tissue. Based on our results we recommend that: (a) tissue samples with RIN < 5 are excluded from analyses; (b) RIN is considered when selecting matched cases; (c) ΔC_T_ correlations between genes of interest and RIN are reported; and (d) RIN is included as a co-variate during statistical analyses.

## Data Availability Statement

The original contributions presented in the study are included in the article/Supplementary Material, further inquiries can be directed to the corresponding author.

## Ethics Statement

This study was reviewed and approved by the University of Auckland Human Participants Ethics Committee (011654). *Post-mortem* human brain tissue was obtained from the Neurological Foundation Human Brain Bank at the University of Auckland. Human tissue was donated to the Brain Bank with consent from donors’ families and its use in this project was approved by the University of Auckland Human Participants Ethics Committee.

## Author Contributions

BH and BR contributed to the conceptualization, methodology, analysis, writing, and editing. RP contributed to the methodology, analysis, and editing. RF contributed to the resources and editing. MC contributed to the resources, analysis, and editing. All authors contributed to the article and approved the submitted version.

## Conflict of Interest

The authors declare that the research was conducted in the absence of any commercial or financial relationships that could be construed as a potential conflict of interest.

## Publisher’s Note

All claims expressed in this article are solely those of the authors and do not necessarily represent those of their affiliated organizations, or those of the publisher, the editors and the reviewers. Any product that may be evaluated in this article, or claim that may be made by its manufacturer, is not guaranteed or endorsed by the publisher.
