# Supplementary material for: RNA Quality in Post-mortem Human Brain Tissue Is Affected by Alzheimer’s Disease
Source: Front Mol Neurosci. 2021 Dec 21;14:780352. doi: 10.3389/fnmol.2021.780352 (PMC8724529; doi:10.3389/fnmol.2021.780352)
Supplement: Supplementary file 1 [file Presentation_1.PPTX]

## Slide 1
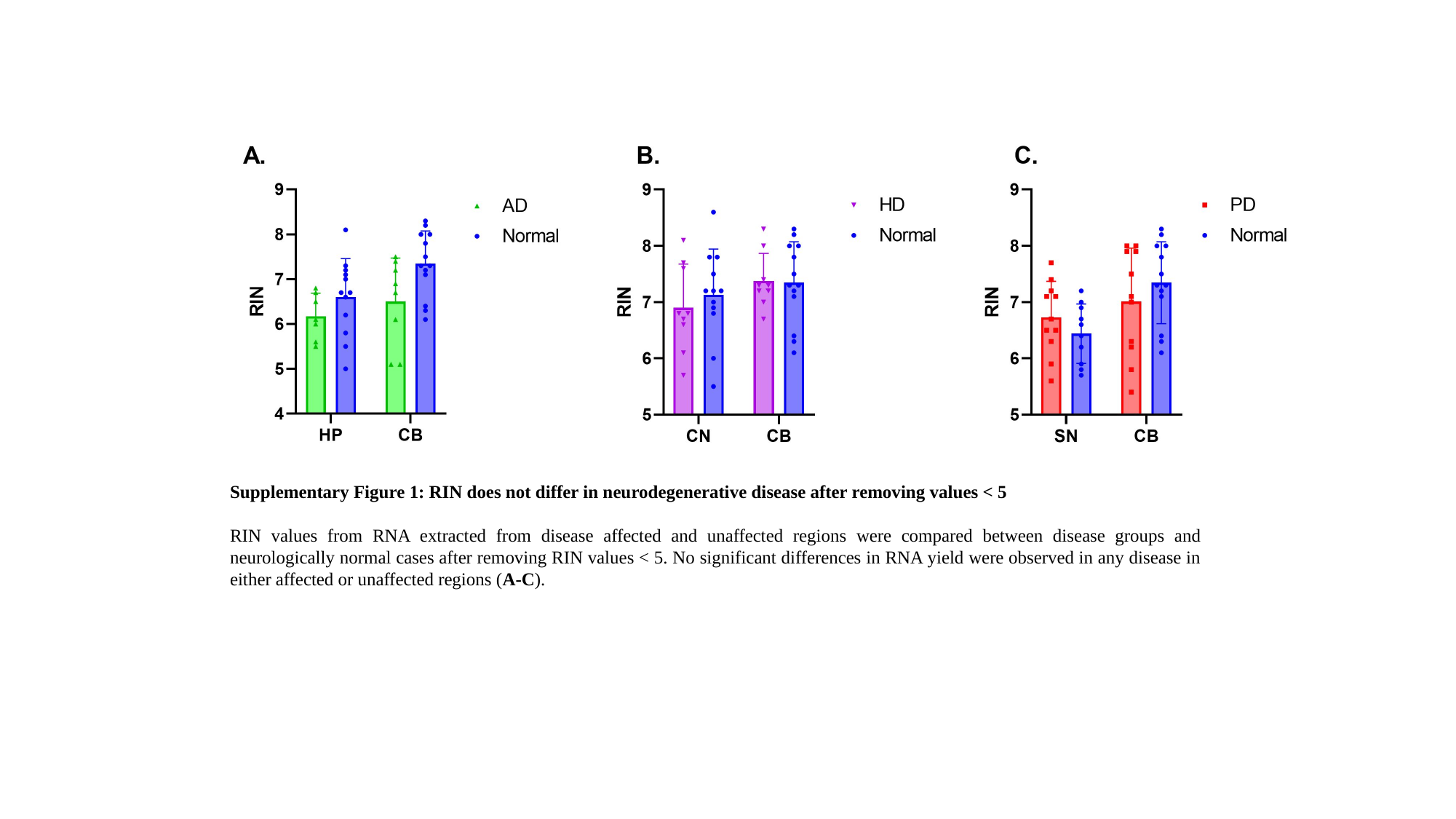

Supplementary Figure 1: RIN does not differ in neurodegenerative disease after removing values < 5
RIN values from RNA extracted from disease affected and unaffected regions were compared between disease groups and neurologically normal cases after removing RIN values < 5. No significant differences in RNA yield were observed in any disease in either affected or unaffected regions (A-C).

## Slide 2
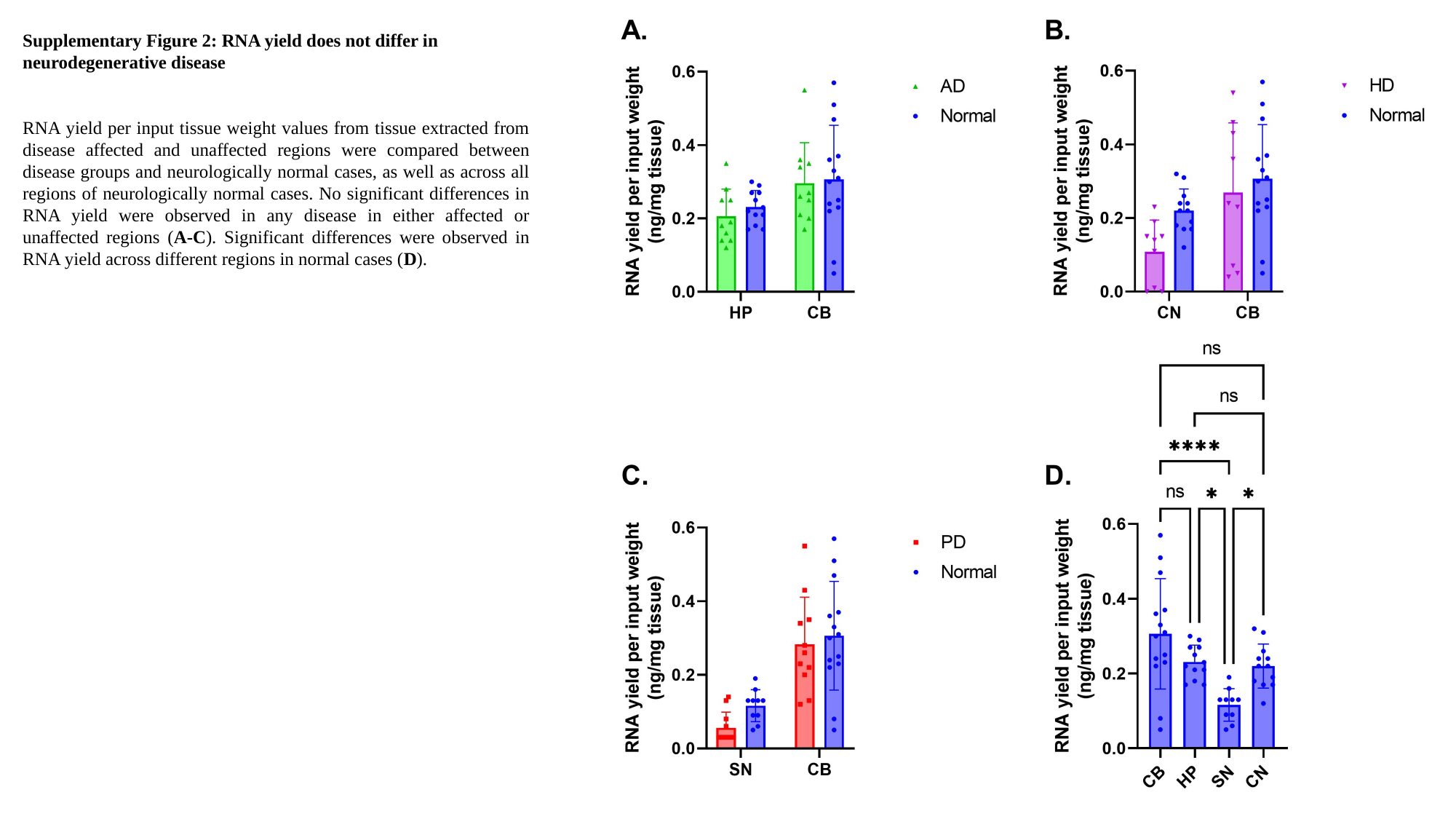

Supplementary Figure 2: RNA yield does not differ in neurodegenerative disease
RNA yield per input tissue weight values from tissue extracted from disease affected and unaffected regions were compared between disease groups and neurologically normal cases, as well as across all regions of neurologically normal cases. No significant differences in RNA yield were observed in any disease in either affected or unaffected regions (A-C). Significant differences were observed in RNA yield across different regions in normal cases (D).

## Slide 3
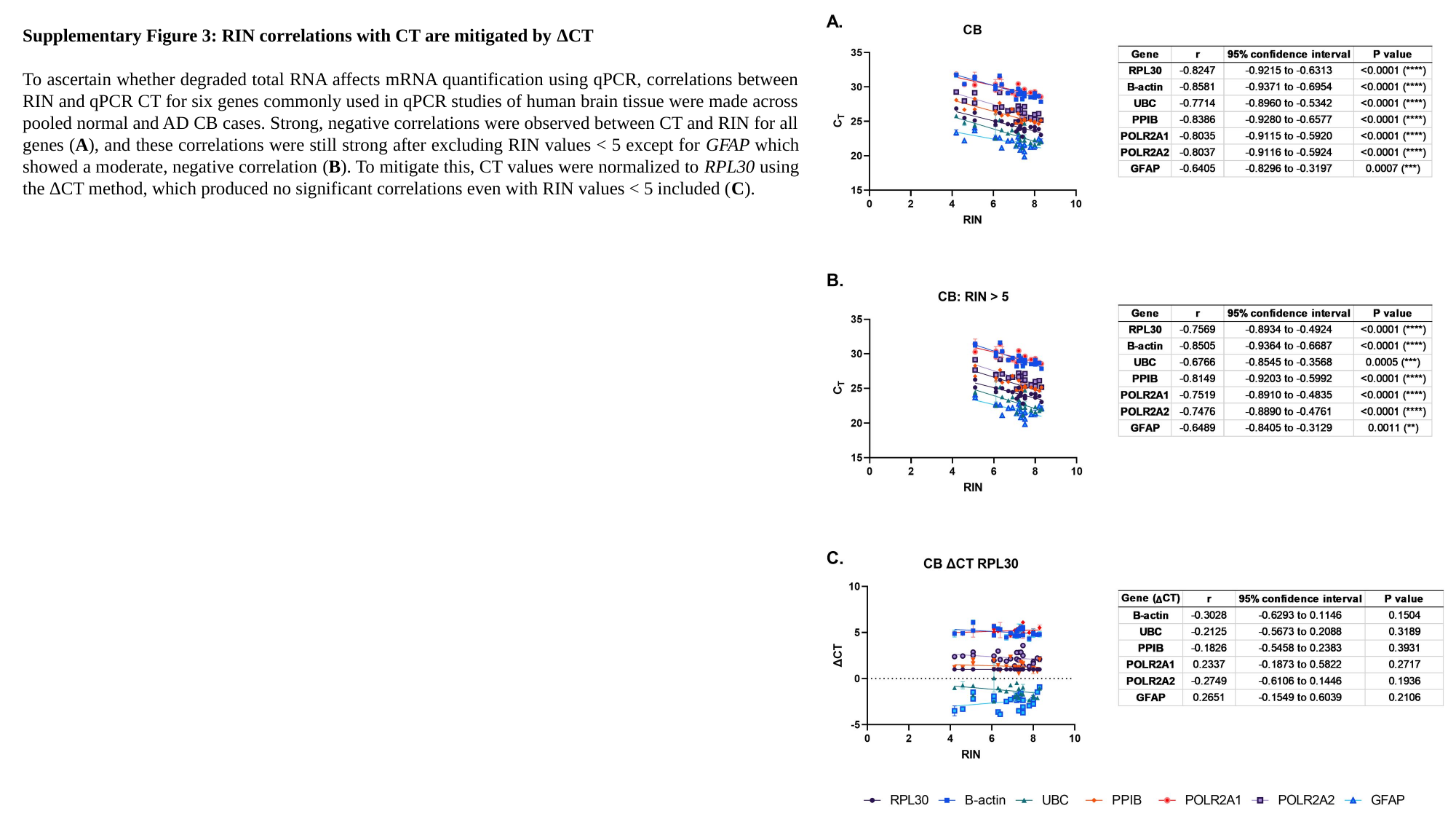

Supplementary Figure 3: RIN correlations with CT are mitigated by ΔCT
To ascertain whether degraded total RNA affects mRNA quantification using qPCR, correlations between RIN and qPCR CT for six genes commonly used in qPCR studies of human brain tissue were made across pooled normal and AD CB cases. Strong, negative correlations were observed between CT and RIN for all genes (A), and these correlations were still strong after excluding RIN values < 5 except for GFAP which showed a moderate, negative correlation (B). To mitigate this, CT values were normalized to RPL30 using the ΔCT method, which produced no significant correlations even with RIN values < 5 included (C).

## Slide 4
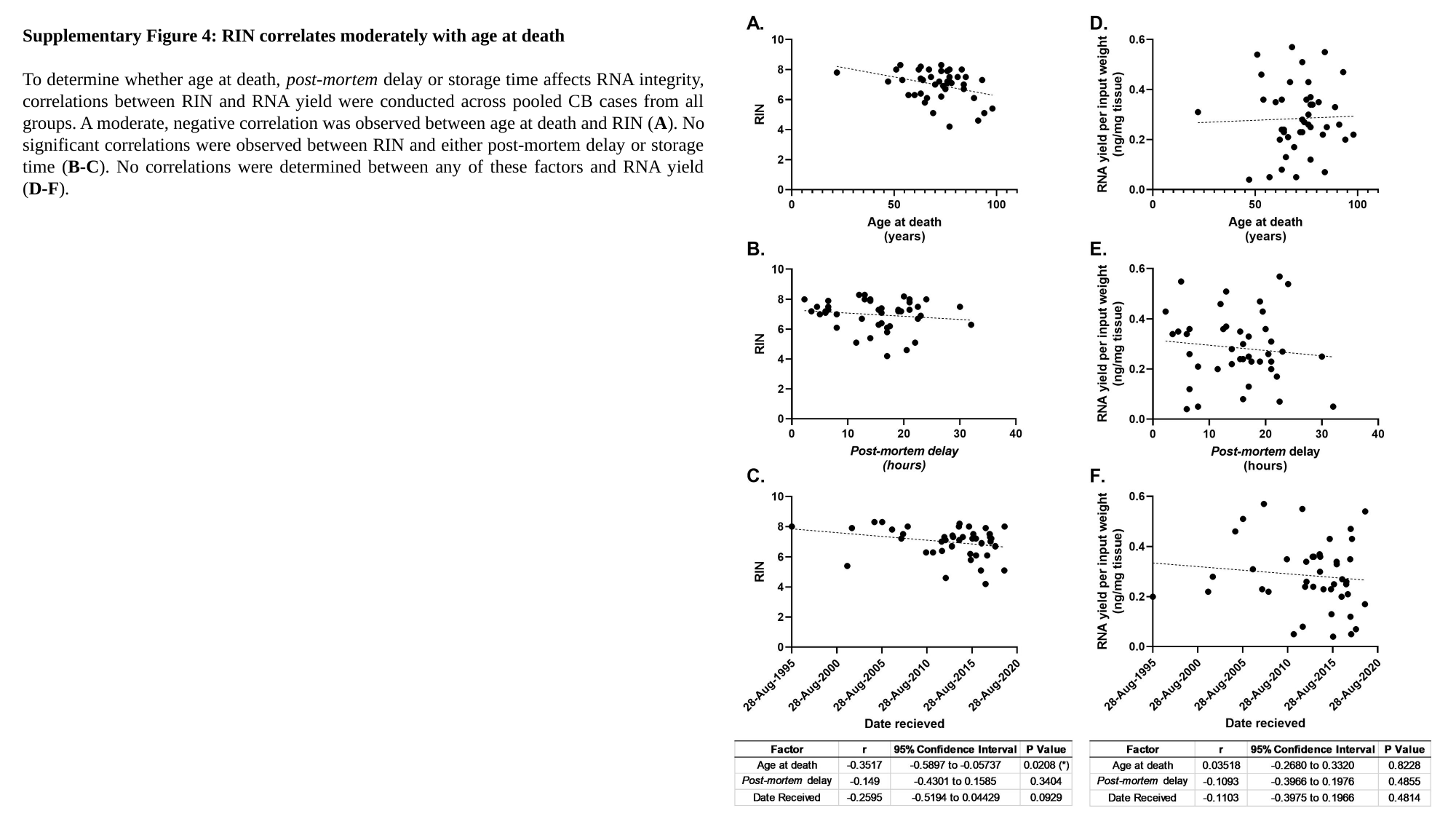

Supplementary Figure 4: RIN correlates moderately with age at death
To determine whether age at death, post-mortem delay or storage time affects RNA integrity, correlations between RIN and RNA yield were conducted across pooled CB cases from all groups. A moderate, negative correlation was observed between age at death and RIN (A). No significant correlations were observed between RIN and either post-mortem delay or storage time (B-C). No correlations were determined between any of these factors and RNA yield (D-F).
